# Ghrelin Protection against Lipopolysaccharide-Induced Gastric Mucosal Cell Apoptosis Involves Constitutive Nitric Oxide Synthase-Mediated Caspase-3 S-Nitrosylation

**DOI:** 10.1155/2010/280464

**Published:** 2010-03-30

**Authors:** Bronislaw L. Slomiany, Amalia Slomiany

**Affiliations:** Research Center, NJ Dental School, University of Medicine and Dentistry of New Jersey, 110 Bergen Street, P.O. Box 1709, Newark, NJ 07103-2400, USA

## Abstract

Ghrelin, a peptide hormone produced mainly in the stomach, has emerged as an important modulator of the inflammatory responses that are of significance to the maintenance of gastric mucosal integrity. Here, we report on the role of ghrelin in controlling the apoptotic processes induced in gastric mucosal cells by *H. pylori* lipopolysaccharide (LPS). The countering effect of ghrelin on the LPS-induced mucosal cell apoptosis was associated with the increase in constitutive nitric oxide synthase (cNOS) activity, and the reduction in caspase-3 and inducible nitric oxide synthase (NOS-2). The loss in countering effect of ghrelin on the LPS-induced changes in apoptosis and caspase-3 activity was attained with Src kinase inhibitor, PP2, as well as Akt inhibitor, SH-5, and cNOS inhibitor, L-NAME. Moreover, the effect of ghrelin on the LPS-induced changes in cNOS activity was reflected in the increased cNOS phosphorylation that was sensitive to SH-5. Furthermore, the ghrelin-induced up-regulation in cNOS activity was associated with the increase in caspase-3 S-nitrosylation that was susceptible to the blockage by L-NAME. Therefore, ghrelin protection of gastric mucosal cells against *H. pylori* LPS-induced apoptosis involves Src/Akt-mediated up-regulation in cNOS activation that leads to the apoptotic signal inhibition through the NO-induced caspase-3 S-nitrosylation.

## 1. Introduction

Lipopolysaccharide (LPS), a component of the outer membrane of Gram-negative bacterium *H. pylori *colonizing the gastric mucosa, is recognized as a potent endotoxin responsible for eliciting mucosal inflammatory responses that characterize gastritis and duodenal ulcers [1, 2]. Indeed, the gastric mucosal response to *H. pylori *LPS or associated with gastritis caused by *H. pylori *infection is manifested by the increase in proinflammatory cytokine production, disturbances in nitric oxide generation system, and a massive rise in epithelial cell apoptosis [1, 3, 4].

Of the three nitric oxide synthase (NOS) isozymes responsible for NO generation, the two expressed constitutively (cNOS) are Ca^2+^-dependent and provide precise pulses of NO for a fine modulation of the cellular processes, including the inhibition of apoptogenic signals [5, 6]. The inducible isoform of NOS, known as iNOS or NOS-2, is Ca^2+^-independent and, once induced, provides a high output of NO generation for host defense. However, its massive and sustained activation may have also cytotoxic consequences, causing transcriptional disturbances and the induction of apoptosis through the activation of a group of aspartate-specific cysteine proteases, caspases [6, 7]. 

Based on their function, the caspase proteases are categorized into initiator and executioner subtypes [6, 8]. The implementation of the apoptotic program requires the participation of both subtypes of caspases, the initiator caspases (caspases-8, -9, and -10), which activate the executioner caspases (caspses-3, -6, and -7), which in turn cleave the targeted intracellular substrates [8, 9]. The activation of executioner caspases is recognized as an irreversible commitment to the execution phase of apoptosis characterized by cytoplasmic shrinkage, breakdown of nuclear envelope, condensation of chromatin structure, and DNA fragmentation [8, 9].

A substantial volume of data indicates that the propagation of apoptogenic signal is influenced by intracellular NO production [6, 7, 10]. Indeed, NO is capable of affecting the function of many proteins by reacting with their cysteine amino acid residue to form S-nitrosothiols, and all caspases are known to contain a critical cysteine at the catalytic site that is a target of S-nitrosylation [7, 10, 11]. Moreover, NO has been shown to possess both the pro- and anti-apoptotic effects depending on cell and type of the NOS isozyme involved. In general, S-nitrosylation resulting form sustained NOS-2 activation appears to have proapoptotic effect, while the low level of NO production by cNOS is believed to be a transient cell-signaling event that has anti-apoptotic effect [5, 7]. Indeed, the inhibition of cNOS has been reported to potentiate ischemia-reperfusion-induced myocardial apoptosis via a caspase-3-dependent pathway, while the inhibition of apoptosis was attained by caspase-3 S-nitrosylation [7, 12].

Recent advances into the nature of factors involved in the maintenance of gastric mucosal integrity that are capable of influencing the extent of mucosal inflammatory responses have resulted in identification of ghrelin [13]. This 28-amino acid peptide hormone, produced mainly in the stomach, has emerged as an important modulator of the processes of gastric mucosal repair, protection against acute mucosal injury by ethanol, and the control of local inflammatory responses to bacterial infection [14–16]. Moreover, ghrelin has been identified as an important regulator of the mucosal NOS system responsible for NO production [17, 18]. 

In this study, we investigated the influence of ghrelin on the apoptotic processes induced in gastric mucosal cells by *H. pylori *lipopolysaccharide. Our data revealed that ghrelin protection of the mucosal cells against the LPS-induced apoptosis results from cNOS-derived NO inhibition of caspase-3 activity through S-nitrosylation.

## 2. Materials and Methods

### 2.1. Mucosal Cell Incubation

The gastric mucosal cells, collected by scraping the mucosa of freshly dissected rat stomachs with a blunt spatula, were suspended in five volumes of ice-cold Dulbecco's modified (Gibco) Eagle's minimal essential medium (DMEM), supplemented with fungizone (50 *μ*g/mL), penicillin (50 U/mL), streptomycin (50 *μ*g/mL), and 10% fetal calf serum, and gently dispersed by trituration with a syringe, and settled by centrifugation [19]. Following rinsing, the cells were resuspended in the medium to a concentration of 2 × 10^7^ cell/mL, transferred in 1 mL aliquots to DMEM in culture dishes, and incubated under 95% O_2_, 5% CO_2_, and atmosphere at 37°C for 16 h in the presence of 0–200 ng/mL of *H. pylori *LPS [20]. In the experiments evaluating the effect of ghrelin (rat, Sigma), cNOS inhibitor, L-NAME, iNOS inhibitor, 1400 W, Src inhibitor, PP2, Akt inhibitor, SH-5 (Calbiochem), and ascorbate (Sigma), the cells were first preincubated for 30 minutes with the indicated dose of the agent or vehicle before the addition of the LPS [20]. The viability of cell preparations before and during the experimentation, assessed by Trypan blue dye exclusion assay [20], was greater than 97%.

### 2.2. Apoptosis and Caspase-3 Activity Assay

For apoptotic measurements, the cells from the control and various experimental conditions were settled by centrifugation, and incubated in the lysis buffer (Boehringer Mannheim). Following centrifugation, the supernatant containing the cytoplasmic histone-associated DNA fragments was reacted with immobilized antihistone antibody. After washing, the retained complex was reacted with anti-DNA peroxidase, and probed with ABTS reagent for spectrophotometric quantification [20]. Caspase-3 activity assays were conducted with using Quanti Zyme assay system (Biomol). The cells from the control and experimental treatments were incubated with the lysis buffer according to the manufacturer's instruction, followed by centrifugation at 10,000 ×g for 10 min. The aliquots of the resulting cytosolic fraction were incubated with 50 *μ*M of Ac-DEVD-pNA substrate for 1 h at 37*º*C, and the caspase-3 activity were measured spectrophotometrically [19].

### 2.3. cNOS and NOS-2 Activity Assay

Nitric oxide synthase activities of cNOS and NOS-2 enzymes in the gastric mucosal cells was measured by monitoring the conversion of L-[^3^H] arginine to L-[^3^H] citrulline using NOS-detect kit (Stratagene). The cells from the control and experimental treatments were homogenized in a sample buffer containing either 10 mM EDTA (for NOS-2) or 6 mM CaCl_2_ (for cNOS), and centrifuged [21]. The aliquots of the resulting supernatant were incubated for 30 minutes at 25°C in the presence of 50 *μ*Ci/mL of L-[^3^H]arginine, 10 mM NAPDH, 5 *μ*M tetrahydrobiopterin, and 50 mM Tis-HCl buffer, pH 7.4, in a final volume of 250 *μ*l. Following addition of stop buffer and Dowex-50 W (Na^+^)resin, the mixtures were transferred to spin cups and centrifuged, and the formed L-[^3^H]citrulline contained in the flow through was quantified by scintillation counting.

### 2.4. Caspase-3 S-Nitrosylation

A biotin-switch procedure was employed to assess caspase-3 protein S-nitrosylation [22, 23]. The mucosal cells, treated with ghrelin (0.5 *μ*g/ml) or L-NAME (300 *μ*M) + ghrelin and incubated for 16h in the presence of 100 ng/ml of *H. pylori *LPS, were lysed in 0.2 mL of HEN lysis buffer, pH 7.7, and the unnitrosylated thiol groups were blocked with S-methyl methanethiosulfonate reagent [23]. The proteins were precipitated with acetone, resuspended in 0.2 mL of HEN buffer containing 1% SDS, and subjected to targeted nitrothiol group reduction with sodium ascorbate (100 mM). The free thiols were then labeled with biotin and the biotinylated proteins were recovered on streptavidin beads. The formed streptavidin bead-protein complex was washed with neutralization buffer, and the bound proteins were dissociated from streptavidin beads with 50 *μ*l of elution buffer (20 mM HEPES, 100 mM NaCl, 1 mM EDTA, pH 7.7) containing 1% 2-mercaptoethanol [23]. The obtained proteins were then analyzed by Western blotting.

### 2.5. Western Blot Analysis

The mucosal cells, collected by centrifugation, were resuspended for 30 minutes in ice-cold lysis buffer [17], and following brief sonication the lysates were centrifuged at 12,000 g for 10 minutes, and the supernatants were subjected to protein determination using BCA protein assay kit (Pierce). The samples, including those subjected to biotin-switch procedure, were then resuspended in loading buffer, boiled for 5 minutes, and subjected to SDS-PAGE using 30 *μ*g protein/lane. The separated proteins were transferred onto nitrocellulose membranes, blocked with 5% skim milk, and probed with the antibody against phosphorylated protein at 4°C for 16 h. After 1h of incubation with the horseradish peroxidase-conjugated secondary antibody, the phosphorylated proteins were revealed using an enhanced chemiluminescence. Membranes were stripped by incubation in 1M Tris-HCl (pH 6.8), 10% SDS, and 10 mM dithiotreitol for 30 minutes at 55°C, and reprobed with antibody against proteins of interest. Immunoblotting was performed using specific antibodies directed against cNOS, phospho-cNOS (Calbiochem), and caspase-3 (Sigma).

### 2.6. Data Analysis

All experiments were carried out using duplicate sampling, and the results are expressed as means ± SD. Analysis of variance (ANOVA) was used to determine significance and the significance level was set at *P* < .05.

## 3. Results

**Figure 1:**
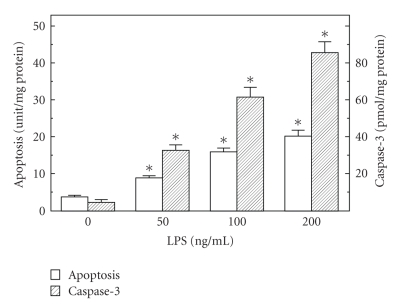
Effect of *H. pylori *LPS on rat gastric mucosal cell apoptosis and caspase-3 activity. The cells were treated with the indicated concentrations of the LPS and incubated for 16 h. Values represent the means ± SD of five experiments. *P < .05 compared with that of control (LPS, 0).

**Figure 2:**
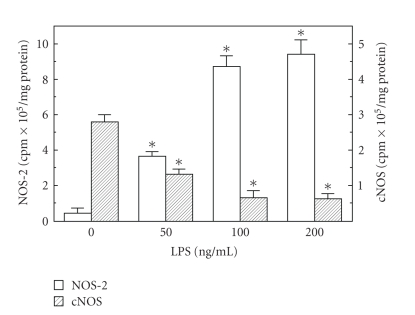
Effect of *H. pylori *LPS on the expression of inducible (NOS-2) and constitutive (cNOS) nitric oxide synthase activities in rat gastric mucosal cells. The cells were treated with the indicated concentrations of the LPS and incubated for 16 h. Values represent the means ± SD of five experiments. *P < .05 compared with that of control (LPS, 0).

The role of ghrelin in modulation of the apoptotic processes associated with *H. pylori *infection was investigated using rat gastric mucosal cells exposed to *H. pylori *key virulence factor, LPS. Employing apoptotic DNA fragmentation assay in conjunction with the measurements of caspase-3 activity, we demonstrated that the LPS caused a dose-dependent increase in gastric mucosal cell apoptosis and caspase-3 activity, which at 100 *μ*g/ml LPS reached respective values of 4.4- and 13.5-fold over that of controls (Figure 1). Moreover, we found that the LPS at 100 *μ*g/ml also caused a 19.8-fold increase in gastric mucosal cell NOS-2 activity, while the cNOS activity showed a 4.3-fold decrease (Figure 2).

**Figure 3:**
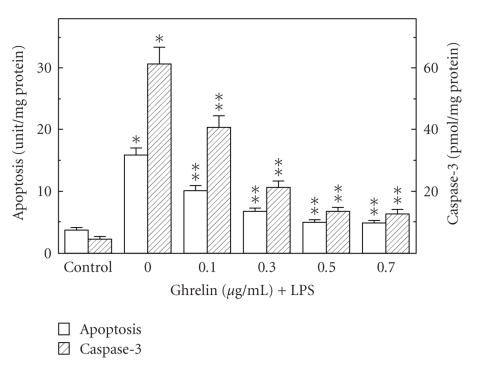
Effect of ghrelin on *H. pylori *LPS-induced gastric mucosal cell apoptosis and the activity of caspase-3. The cells, preincubated with the indicated concentrations of ghrelin, were treated with the LPS at 100 ng/mL and incubated for 16 h. Values represent the means ± SD of five experiments. *P < .05 compared with that of control. **P < .05 compared with that of LPS alone.

**Figure 4:**
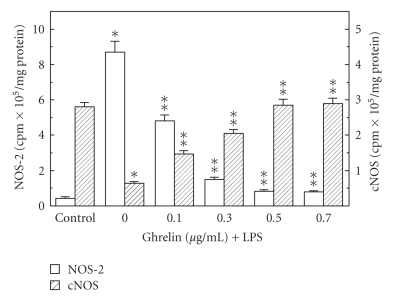
Effect of ghrelin on *H. pylori *LPS-induced expression of NOS-2 and cNOS activities in gastric mucosal cells. The cells, preincubated with the indicated concentrations of ghrelin, were treated with the LPS at 100 ng/mL and incubated for 16 h. Values represent the means ± SD of five experiments. *P < .05 compared with that of control. **P < .05 compared with that of LPS alone.

**Figure 5:**
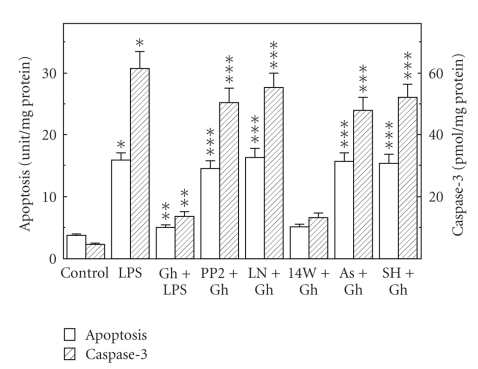
Effect of nitric oxide synthase inhibitors on the ghrelin (Gh-) induced changes in gastric mucosal cell apoptosis and the activity of caspase-3. The cells, preincubated with 30 *μ*M PP2, 300 *μ*M L-NAME (LN), 20 *μ*M 1400 W (14 W), 300 *μ*M ascorbate (As), or 20 *μ*M SH-5 (SH), were treated with Gh at 0.5 *μ*g/mL and incubated for 16 h in the presence of 100 ng/mL of *H. pylori *LPS. Values represent the means ± SD of five experiments. *P < .05 compared with that of control. **P < .05 compared with that of LPS alone. ***P < .05 compared with that of Gh*+*LPS.

**Figure 6:**
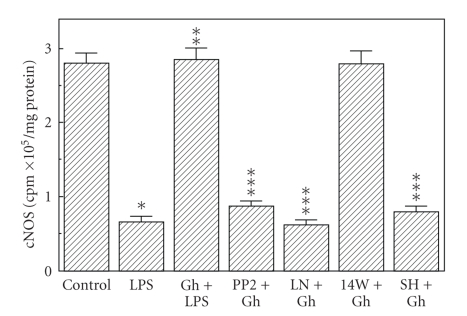
Effect of nitric oxide synthase inhibitors on the ghrelin (Gh-) induced changes in cNOS activity in gastric mucosal cell exposed to *H. pylori *LPS. The cells, preincubated with 30 *μ*M PP2, 300 *μ*M L-NAME (LN), 20 *μ*M 1400 W (14 W), or 20 *μ*M SH-5 (SH), were treated with Gh at 0.5 *μ*g/mL and incubated for 16 h in the presence of 100 ng/mL LPS. Values represent the means ± SD of five experiments. *P < .05 compared with that of control. **P < .05 compared with that of LPS alone. ***P < .05 compared with that of Gh*+*LPS.

Preincubation of the mucosal cells with ghrelin led to a concentration-dependent decrease in the LPS-induced changes, and at the optimal concentration of 0.5 *μ*g/ml resulted in a 68.7% drop in apoptosis and a 78% reduction in caspase-3 activity (Figure 3), as well as a 90.2% decrease in NOS-2 activity (Figure 4). However, the activity of gastric mucosal cell cNOS increased by a 77.5% (Figure 4). Further, we found that significant loss in the preventive effect to ghrelin on the LPS-induced changes in the mucosal cell apoptosis and caspase-3 activity occurred with Src kinase inhibitor, PP2, as well as Akt inhibitor, SH-5, and cNOS inhibitor, L-NAME, while selective NOS-2 inhibitor, 1400 W, had no effect (Figure 5). Moreover, the effects PP2 and SH-5, like that of L-NAME, were reflected in the inhibition of ghrelin-induced cNOS activity (Figure 6). 

**Figure 7:**
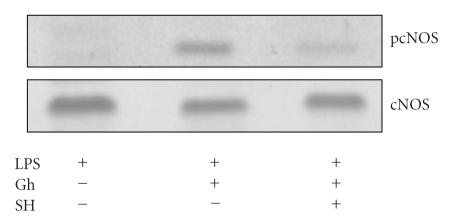
Effect of Akt inhibitor, SH-5 (SH), on ghrelin- (Gh-) induced cNOS phosphorylation in gastric mucosal cells exposed to *H. pylori *LPS. The cells were treated with Gh (0.5 *μ*g/mL) or SH (20 *μ*M) + Gh and incubated for 16 h in the presence of 100 ng/ml LPS. Cell lysates were resolved on SDS-PAGE, transferred to nitrocellulose, and probed with phosphorylation-specific cNOS (pcNOS) antibody, and after stripping reprobed with anti-cNOS antibody. The immunoblots shown are representative of three experiments.

To gain additional leads into the mechanism of ghrelin-induced signaling resulting in up-regulation in gastric mucosal cell cNOS activity, we examined the effect of ghrelin on the cNOS phosphorylation. As cNOS is known to undergo a rapid posttranslational activation through phosphorylation at Ser^1177^ by kinase Akt [17, 18], the cells prior to ghrelin incubation were pretreated with Akt inhibitor, SH-5, and the lysates were examined for cNOS activation using antibody directed against total cNOS and phosphorylated cNOS (pcNOS). As shown in Figure 7, the countering effect of ghrelin on the LPS-induced changes in the mucosal cell cNOS activity was reflected in a marked increase in the enzyme protein phosphorylation, while the suppression of ghrelin effect by Akt inhibitor, SH-5, was manifested in a drop in the cNOS phosphorylation. 

**Figure 8:**
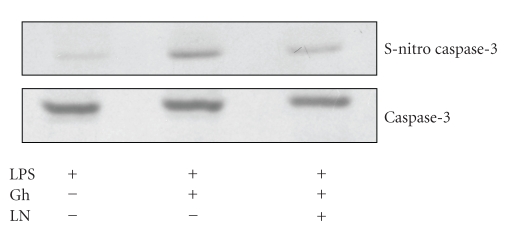
Effect of cNOS inhibitor, L-NAME (LN), on ghrelin- (Gh-) induced caspase-3 S-nitrosylation in gastric mucosal cells exposed to *H. pylori *LPS. The cells were treated with Gh (0.5 *μ*g/mL) or LN (300 *μ*M) + Gh and incubated for 16 h in the presence of 100 ng/mL LPS. A portion of the cell lysates was processed by biotin-switch procedure for protein S-nitrosylation and, along with the reminder of the lysates, subjected to SDS-PAGE, transferred to nitrocellulose, and probed with anti-caspase-3 antibody. The immunoblots shown are representative of three experiments.

Since NO is known to exert the modulatory effect on the apoptotic processes through caspase cysteine S-nitrosylation [6, 7, 12], we next analyzed the influence of ghrelin on the mucosal cell caspase-3 S-nitrosylation. The results revealed that ghrelin countering effect on the LPS-induced up-regulation in the mucosal cell apoptosis and caspase-3 activity was susceptible to suppression by ascorbate (Figure 5), which is in keeping with well-known susceptibility of S-nitrosylated proteins to this reducing agent [17, 22, 23]. Furthermore, Western blot analysis of the cell lysates subjected to biotin-switch procedure and probing with antibody against caspase-3 revealed that ghrelin countering effect on the LPS-induced up-regulation in the caspase-3 activity was manifested in the increase in caspase-3 S-nitrosylation. Preincubation with L-NAME on the other hand caused the blockage in the ghrelin-induced caspase-3 S-nitrosylation (Figure 8). Collectively, these data demonstrate that ghrelin protection of gastric mucosal cells against *H. pylori *LPS-induced apoptosis involves cNOS-induced suppression of caspase-3 activity through S-nitrosylation. 

## 4. Discussion

Nitric oxide, a gaseous signaling molecule, is recognized as an important effector of a wide variety of regulatory pathways that are of significance to cellular survival and the inflammatory responses to bacterial infection. Moreover, due to its high reactivity, NO is also capable of affecting the function of a number of proteins by reacting with cysteine residues to form S-nitrosothiols [7, 10, 12]. Rapidly accumulating evidence suggests that, like posttranslational modification through phosphorylation, the protein S-nitrosylation is a targeted and reversible posttranslational modification that regulates protein activity during cell signaling [6, 7, 11]. Indeed, S-nitrosylation of mitochondrial protein thiols is known to block cell death after glutathione depletion, and contributes to redox regulatory activity and antiapoptotic function of thioredoxin, and S-nitrosylation of a key executioner caspase, caspase-3, has been implicated in the regulation of apoptotic processes by the NOS enzyme complex [7, 10, 12, 24]. Moreover, it became apparent that pro- or antiapoptotic effects of NO-induced S-nitrosylation depend upon the targeted protein colocalization with the NOS-2 or cNOS isozyme system [7, 10].

Hence, in keeping with recent evidence for the role of ghrelin in controlling gastric mucosal inflammatory responses through regulation of the mucosal NO production [15–18], in the present study we examined the influence of this peptide hormone on the apoptotic processes associated with *H. pylori *infection. Using rat gastric mucosal cells exposed to *H. pylori *key virulence factor, LPS, we demonstrated that the LPS-induced enhancement in the mucosal cell apoptosis and caspase-3 activity was associated with a marked decrease in cNOS activity and up-regulation in NOS-2. Further, our results revealed that the countering effect of ghrelin on the LPS-induced mucosal cell apoptosis was reflected in the increase in the cNOS activity, and the reduction in caspase-3 and NOS-2 activity. Moreover, a significant loss in the countering effect of ghrelin on the LPS-induced changes in the mucosal cell apoptosis and caspase-3 activity was attained with cNOS inhibitor, L-NAME, whereas selective NOS-2 inhibitor, 1400 W, had no effect. These findings are thus in keeping with the results of earlier studies demonstrating that the proapoptotic effects of *H. pylori *on gastric epithelial cell integrity relay on NOS-2 participation in the amplification of the cell death signaling cascade [4, 21]. The fact that the induced proapoptotic events were also associated with a marked decrease in cNOS activity, while the countering effects of ghrelin were reflected in a decrease in NOS-2 and up-regulation in cNOS activities, furthermore attests to the modulatory influence of cNOS on the LPS-induced apoptogenic signal propagation. 

Further, we found that the countering effect of ghrelin on the LPS-induced changes in gastric mucosal cell apoptosis and caspase-3 activity were subject to suppression by Src kinase inhibitor, PP2, as well as Akt inhibitor, SH-5; both of which also abrogated the ghrelin-induced up-regulation in cNOS activity. Hence, we concluded that ghrelin countering effect on the LPS-induced proapoptotic events occurs with the involvement of Src-kinase-mediated cNOS activation through Akt. Our assertion is supported by the data showing that the countering effect of ghrelin on the LPS-induced changes in cNOS activity was reflected in the increase in enzyme protein phosphorylation, while the suppression of ghrelin effect by Akt inhibitor, SH-5, was manifested in a drop in cNOS phosphorylation. Indeed, recent reports show that posttranslational regulation of cNOS activity involves a rapid enzyme protein phosphorylation at the critical Ser^1177^ with the participation of Src/Akt pathway, and that ghrelin induces up-regulation in NO production by the activation of Akt-mediated cNOS phosphorylation [17, 18, 25].

A substantial volume of evidence implicates NO in the regulation of apoptotic cell death through the modulation of apoptogenic signals propagated by the caspase cascade [6, 7, 10]. Upon apoptotic stimulation, these cysteine aspartyl proteases are known to undergo NO-induced targeted S-nitrosylation with pro- or antiapoptotic consequences, depending on subcellular localization and the proximity to NOS-2 or cNOS isozyme system [10, 11]. In our study, we assessed the influence of ghrelin on S-nitrosylation of the key executioner caspase, caspase-3. We found that, in keeping with well-known vulnerability of S-nitrosylated proteins to reduction by ascorbic acid [17, 22, 23], the ghrelin countering effect on *H. pylori *LPS-induced up-regulation in gastric mucosal cell caspase-3 activity showed susceptibility to the suppression by ascorbate. Moreover, Western blot analysis of the mucosal cell lysates subjected to biotin-switch procedure revealed that ghrelin countering effect on the LPS-induced up-regulation in caspase-3 activity was reflected in the increased caspse-3 S-nitrosylation. The suppression of ghrelin effect on NO production with cNOS inhibitor, L-NAME, led to the blockage of caspase-3 S-nitrosylation, thus supporting the role of cNOS-induced caspase-3 S-nitrosylation in the mechanism of ghrelin protection against *H. pylori-*induced gastric mucosal cell apoptosis. 

In summary, we provide evidence that ghrelin is capable of gastric mucosal protection against *H. pylori *LPS-induced apoptosis. The data suggest that the modulatory effect of ghrelin occurs with the involvement of Src/Akt kinase-mediated up-regulation in cNOS activation through phosphorylation, and that cNOS-mediated caspase-3 S-nitrosylation induced by ghrelin plays an essential role in the apoptotic signal cascade inhibition.

## References

[1] Piotrowski J, Piotrowski E, Skrodzka D, Slomiany A, Slomiany BL (1997). Induction of acute gastritis and epithelial cell apoptosis by *H. pylori* lipopolysaccharide. *Scandinavian Journal of Gastroenterology*.

[2] de Boer WA (2000). Topics in *Helicobacter pylori* infection: focus on a ‘search-and-treat’ strategy for ulcer disease. *Scandinavian Journal of Gastroenterology*.

[3] Fu S, Ramanujam KS, Wong A (1999). Increased expression and cellular localization of inducible nitric oxide synthase and cyclooxygenase 2 in *Helicobacter pylori* gastritis. *Gastroenterology*.

[4] Slomiany BL, Slomiany A (2001). Blockade of p38 mitogen-activated protein kinase pathway inhibits inducible nitric oxide synthase and gastric mucosal inflammatory reaction to *Helicobacter pylori* lipopolysaccharide by peroxisome proliferator-activated receptor *γ* activation. *Inflammopharmacology*.

[5] Kim Y-M, Talanian RV, Billiar TR (1997). Nitric oxide inhibits apoptosis by preventing increases in caspase-3-like activity via two distinct mechanisms. *Journal of Biological Chemistry*.

[6] Mannick JB, Schonhoff C, Papeta N (2001). S-nitrosylation of mitochondrial caspases. *Journal of Cell Biology*.

[7] Sun J, Steenbergen C, Murphy E (2006). S-nitrosylation: NO-related redox signaling to protect against oxidative stress. *Antioxidants and Redox Signaling*.

[8] Thornberry NA, Lazebnik Y (1998). Caspases: enemies within. *Science*.

[9] Reed JC (1997). Cytochrome c: can’t live with it—can’t live without it. *Cell*.

[10] Mannick JB (2007). Regulation of apoptosis by protein S-nitrosylation. *Amino Acids*.

[11] Raines KW, Cao G-L, Lee EK, Rosen GM, Shapiro P (2006). Neuronal nitric oxide synthase-induced S-nitrosylation of H-Ras inhibits calcium ionophore-mediated extracellular-signal-regulated kinase activity. *Biochemical Journal*.

[12] Maejima Y, Adachi S, Morikawa K, Ito H, Isobe M (2005). Nitric oxide inhibits myocardial apoptosis by preventing caspase-3 activity via S-nitrosylation. *Journal of Molecular and Cellular Cardiology*.

[13] Kojima M, Hosoda H, Date Y, Nakazato M, Matsuo H, Kangawa K (1999). Ghrelin is a growth-hormone-releasing acylated peptide from stomach. *Nature*.

[14] Brzozowski T, Konturek PC, Konturek SJ (2004). Exogenous and endogenous ghrelin in gastroprotection against stress-induced gastric damage. *Regulatory Peptides*.

[15] Sibilia V, Pagani F, Rindi G (2008). Central ghrelin gastroprotection involves nitric oxide/prostaglandin cross-talk. *British Journal of Pharmacology*.

[16] Waseem T, Duxbury M, Ito H, Ashley SW, Robinson MK (2008). Exogenous ghrelin modulates release of pro-inflammatory and anti-inflammatory cytokines in LPS-stimulated macrophages through distinct signaling pathways. *Surgery*.

[17] Slomiany BL, Slomiany A (2009). Involvement of constitutive nitric oxide synthase in ghrelin-induced cytosolic phospholipase A_2_ activation in gastric mucosal cell protection against ethanol cytotoxicity. *Inflammopharmacology*.

[18] Xu X, Jhun BS, Ha CH, Jin Z-G (2008). Molecular mechanisms of ghrelin-mediated endothelial nitric oxide synthase activation. *Endocrinology*.

[19] Slomiany BL, Slomiany A (2002). Nitric oxide as a modulator of gastric mucin synthesis: role of ERK and p38 mitogen-activated protein kinase activation. *IUBMB Life*.

[20] Slomiany BL, Slomiany A (2004). Platelet-activating factor mediates *Helicobacter pylori* lipopolysaccharide interference with gastric mucin synthesis. *IUBMB Life*.

[21] Slomiany BL, Piotrowski J, Slomiany A (1999). Gastric mucosal inflammatory responses to *Helicobacter pylori* lipopolysaccharide; down-regulation of nitric oxide synthase-2 and caspase-3 by sulglycotide. *Biochemical and Biophysical Research Communications*.

[22] Jaffrey SR, Erdjument-Bromage H, Ferris D, Tempst P, Snyder SH (2001). Protein S-nitrosylation: a physiological signal for neuronal nitric acid. *Nature Cell Biology*.

[23] Forrester MT, Foster MW, Stamler JS (2007). Assessment and application of the biotin switch technique for examining protein S-nitrosylation under conditions of pharmacologically induced oxidative stress. *Journal of Biological Chemistry*.

[24] Whiteman M, Chua YL, Zhang D, Duan W, Liou Y-C, Armstrong JS (2006). Nitric oxide protects against mitochondrial permeabilization induced by glutathione depletion: role of S-nitrosylation. *Biochemical and Biophysical Research Communications*.

[25] Vecchione C, Maffei A, Collela S (2002). Leptin effect on endothelial nitric oxide synthase is mediated through Akt-endothelial nitric oxide synthase phosphorylation pathway. *Diabetes*.

